# Hsa_circ_0006837 suppresses gastric cancer cell proliferation, migration, and invasion via the modulation of miR-424-5p

**DOI:** 10.1186/s41065-025-00449-w

**Published:** 2025-05-22

**Authors:** Yanxin He, Yeyu Sun, Yinglan Zheng, Yanfang Jiang, Na Li, Wenjie Zhao, Wanhua Ren

**Affiliations:** 1 Department of Gastroenterology, Qingdao Central Hospital, University of Health and Rehabilitation Sciences (Qingdao Central Hospital), Qingdao, 266042 China; 2 Qingdao University, Qingdao, 266042 China; 3 Department of Gastroenterology, Shandong Provincial Hospital, No. 324, Jingwuweiqi Road, Jinan, 250021 China

**Keywords:** Hsa_circ_0006837, Gastric cancer, Prognosis, Biological function, miR-424-5p

## Abstract

**Background:**

The mechanism by which circRERE (hsa_circ_0006837) modulates the malignant progression of gastric cancer was investigated to identify a novel biomarker and therapeutic target for this disease.

**Methods:**

Hsa_circ_0006837 expression in GC tissues and cells was detected by RT-qPCR. Several data analysis methods were used to evaluate the significance of dysregulated hsa_circ_0006837 in GC. The patients were followed up for five years, and survival analysis was conducted using Kaplan-Meier curves. Cox regression was subsequently performed to analyze the risk factors for prognosis. The malignant behaviors of the cells were detected by the CCK-8 and Transwell assays. The relationship between hsa_circ_0006837 and miR-424-5p was assessed by conducting Spearman correlation analysis and verified by dual-luciferase reporter assay.

**Results:**

Hsa_circ_0006837 expression decreased in patients with GC, indicating a poorer patient prognosis. In GC cells, hsa_circ_0006837 overexpression suppressed malignant behaviors. Mechanistically, miR-424-5p was identified as a target of hsa_circ_0006837. The overexpression of miR-424-5p partially counteracted the suppressive effects of upregulated hsa_circ_0006837 on the malignant behaviors of GC cells. FBXO21 was identified as a downstream gene of the hsa_circ_0006837/miR-424-5p axis.

**Conclusions:**

To summarize, hsa_circ_0006837 is a biomarker for the prognosis of GC. Mechanistically, hsa_circ_0006837 overexpression can modulate the malignant behaviors of GC cells through miR-424-5p.

**Supplementary Information:**

The online version contains supplementary material available at 10.1186/s41065-025-00449-w.

## Background

Gastric cancer (GC) greatly contributes to the global burden of cancer. Although the mortality rate has decreased with the advancement of medical practice and technology, GC remains a highly lethal disease due to late diagnosis, with a five-year survival rate of less than 30% [1–3]. The treatment methods for GC mainly include surgery, radiotherapy, and chemotherapy [4, 5]. The prognosis for patients diagnosed with early-stage GC is favorable since early detection and treatment can effectively improve the survival chances of patients with GC. Malignant features such as tumor invasion and metastasis frequently lead to treatment failure in GC patients. Effective monitoring of tumor progression is expected to improve patient prognosis [6]. Therefore, identifying biomarkers that can modulate GC progression and predict patient prognosis is important.

Circular RNAs (circRNAs) are single-stranded RNAs with a closed-loop structure and are noncoding [7]. CircRNAs are not easily digested by exonucleases because of the closed structure formed by back-splicing [8]. Research on the role of circRNAs in diseases has increased in recent years [9]. Several studies have shown that circRNAs function as tumor suppressors or oncogenes in malignant tumors and participate in tumorigenesis and development [10]. Various circRNAs participate in the occurrence and development of GC. Circ-SFMBT2 was found to have a negative regulatory effect on GC development in vitro [11]. By conducting microarray analysis, Zhang et al. reported that the circRNA arginine-glutamic acid dipeptide repeats (circRERE, hsa_circ_0006837) were aberrantly expressed in GC and predicted that hsa_circ_0006837 might bind to miR-424-5p [12]. In a study, circRERE (hsa_circ_0009581) was shown to be helpful in the diagnosis of multiple myeloma and in predicting patient prognosis [13]. CircRERE (hsa_circ_12952) may also serve as a biomarker for colorectal cancer and suppress tumor progression [14], but its clinical significance and biological function in GC need to be investigated. Additionally, the involvement of miR-424-5p in the mechanism underlying the proliferation and invasion of GC cells has been reported in other studies [15]. However, whether hsa_circ_0006837 affects GC cells through the regulation of miR-424-5p needs to be established.

In this study, we determined the significance and functional mechanism of hsa_circ_0006837 in GC by conducting several clinical data analyses and in vitro experiments to identify a novel prognostic and progression biomarker for GC.

## Methods

### Study subjects

The GC and adjacent normal tissue samples used in the study were obtained from 121 GC patients who underwent surgery between April 2016 and February 2018 at Qingdao Central Hospital. The collected samples were rapidly frozen in liquid nitrogen and stored at − 80 °C. The inclusion criteria were as follows: (1) no preoperative radiotherapy, chemotherapy, or immunotherapy; (2) a pathological section of the tissue was examined for GC, and adjacent normal tissues were histologically verified to be free of malignancy; (3) complete clinicopathological data (sex, age, tumor size, etc.); (4) available histopathological samples. The exclusion criteria were as follows: (1) combination of other malignancies and (2) absence of clinicopathological information. All participants provided informed consent. The acquisition of samples and clinical information was permitted by the Ethics Committee of Qingdao Central Hospital. The procedures used in this study adhered to the tenets of the Declaration of Helsinki. The survival status of the GC patients was continuously monitored after surgery, with a follow-up period of 3–60 months.

### Cell culture

GC cells (HGC27, MKN74, and AGS) and normal gastric cells (GES-1) were purchased from ATCC (USA). The cells were grown in DMEM (containing 10% FBS and 1% penicillin-streptomycin) and cultured at 37 °C with 5% CO_2_.

### Cell transfection

The miR-424-5p mimic, miR-424-5p inhibitor, and negative control (50 nM) were obtained from RiboBio (Guangzhou, China). We constructed pcDNA3.1-hsa_circ_0006837 (oe-circ, 50 ng/mL) to upregulate hsa_circ_0006837, with the pcDNA3.1(+) circRNA mini vector (NovoPro, Shanghai, China) used as a negative control (oe NC, 50 ng/mL). Primer sequences for the construction of overexpression vectors are shown in Table S1. Reverse complementary sequences (Alu repeat sequence) that flank the polyclonal site promote back-splicing and cyclization of hsa_circ_0006837. Hsa_circ_0006837 formed by back-splicing of exons 6–11 of the RERE gene. The sequence and structure of hsa_circ_0006837 are shown in the supplementary material (Figure S1). Small interfering RNA (siRNA) targeting FBXO21 (si-FBXO21, 30 nM) and its negative control (si NC, 30 nM) were synthesized by Sangon Biotech (Shanghai, China). The sense-strand sequences of si-FBXO21 were 5’-CAUGAUAAUGGAAAUAGAACU-3’. For the transfection of the above oligonucleotides and plasmids, Lipofectamine 3000 (Invitrogen, USA) was used. The cells were transfected when the cell density was about 60%. Untransfected MKN74 and HGC27 cells were used as the blank group. The cells were collected 24–48 h later for the following experiments.

### RNA extraction and RT-qPCR

The RNA from the tissue homogenates and cells was isolated with TRIzol reagent (Invitrogen, USA) before its purity and concentration were determined. The tissue was mixed with TRIzol, homogenized for 1–2 min, and then lysed for 10 min. The mixture was supplemented with chloroform (chloroform: TRIzol = 1:5), shaken well, and left undisturbed for 10 min. The supernatant was collected in another RNase-free centrifuge tube after the mixture was centrifuged at 12,000 rpm for 15 min (4 °C). The tube was supplemented with isopropanol (isopropanol: TRIzol = 1:2), mixed thoroughly, and left undisturbed for 5–10 min. The supernatant was discarded after centrifugation at 12,000 rpm for 10 min (4 °C). The RNA was washed with 75% ethanol (RNase-free, 75% ethanol: TRIzol = 1:1), and the precipitate was suspended by gentle shaking. The supernatant was discarded after centrifugation at 8,000 rpm for 5 min (4 °C). The RNA sample was dried for 3–5 min and redissolved in 50 µL of RNase-free H_2_O. To extract RNA from cells, the cells were lysed by adding TRIzol and left undisturbed for 10 min. The remaining steps were performed as described above. Finally, the concentration and purity of the extracted RNA were measured.

The extracted RNA was then reverse-transcribed to cDNA using a RevertAid RT Kit to prepare the templates for RT-qPCR. Primers were used to specifically amplify the target transcripts on the CFX96 RT-qPCR system (Bio-Rad, USA) with SYBR Premix Ex Taq (TaKaRa, Japan). Primer sequences for the assay are shown in Table S2. The data were further analyzed using the 2^–ΔΔCt^ method and normalized to β-actin (for hsa_circ_0006837 and FBXO21) and U6 (for miR-424-5p). At least three biological replicates were set up for each experiment.

### Cell proliferation

Cell Counting Kit-8 (CCK-8) reagent (Dojindo, Japan) was used to assess the viability of MKN74 and HGC27 cells in each group at 24, 48, and 72 h of transfection. The reagent contains WST-8, which is reduced to a highly water-soluble yellow formazan by dehydrogenase in cell mitochondria in the presence of the electron carrier 1-methoxy PMS. The quantity of formazan produced is directly proportional to the number of viable cells, and the light absorption value is determined by a microplate reader at 450 nm, which indirectly indicates cell proliferation. The transfected MKN74 and HGC27 cells were incubated in 96-well plates (2 × 10^3^ cells/well) at 37 ℃. Next, 100 µL of RPMI-1640 medium (containing 10 µL of CCK-8) was added to each well at each time point, and incubation was continued for 1 h. After incubation, the absorbance (OD) values of the MKN74 and HGC27 cells were recorded at 450 nm using an Infinite 200 PRO Enzyme Labeler (TECAN, Switzerland), and cell viability was calculated by conducting repeated-measures ANOVA. At least three biological replicates were set up for each experiment.

### Transwell assay

The cell suspension was prepared using an FBS-free medium, and 1 × 10^5^ cells were seeded in the top part of a Matrigel-coated Transwell. Simultaneously, a culture medium supplemented with FBS was added to the bottom chamber. After incubation for 48 h, the invading cells were treated with 4% paraformaldehyde for 20 min. After that, the cells were stained with 0.1% crystal violet for 10 min and then observed under an IX83 inverted microscope (Olympus, Japan). The invading cells were counted and photographed to assess their invasive ability. The migration experiments were performed without Matrigel, and the remaining steps were the same. At least three biological replicates were set up for each experiment.

### Bioinformatics analysis

As predicted by the starBase database (https://rnasysu.com/encori/) [16], miR-424-5p may have binding sites with hsa_circ_0006837. The downstream genes of miR-424-5p were predicted by the databases TargetScanHuman (cumulative weighted context + + score ≤ − 0.5, https://www.targetscan.org/vert_72/) [17], starBase, miRDB (target score ≥ 90, https://mirdb.org/mirdb/index.html) [18], and miRWalk (energy ≤ − 21, http://mirwalk.umm.uni-heidelberg.de/) [19]. The prediction results were visualized with a Venn diagram using the Bioinformatics online platform (https://www.bioinformatics.com.cn/) [20]. The TargetScanHuman database was used to predict binding sites for miR-424-5p and FBXO21. The association of FBXO21 with survival in GC patients was analyzed using the Kaplan-Meier plotter (https://kmplot.com/analysis/) [21]. The expression range of the probes was between 584 and 4353, with a threshold value of 1490 used for the analysis.

### Dual-luciferase reporter assay

The targeting sequences of miR-424-5p in the hsa_circ_0006837 (582 bp) and FBXO21 3’-UTR (428 bp), as well as their mutants, were inserted into the pGL3 vector to construct the luciferase reporter plasmids hsa_circ_0006837-WT (circ-WT), hsa_circ_0006837-MUT (circ-MUT), FBXO21-WT, and FBXO21-MUT. Primer sequences for constructing the reporter vectors are shown in Table S1. The pGL3 vector was used as a negative control. The cells were seeded into in 24-well plates. When the cells reached about 60% confluence, the luciferase reporter vectors (50 ng/mL) were co-transfected with the miR-424-5p mimic (5’-CAGCAGCAAUUCAUGUUUUGAA-3’), inhibitor (5’-UUCAAAACAUGAAUUGCUGC-3’), or their negative controls (50 nM). Untransfected GC cells served as the blank group. The culture was continued for 48 h. Luciferase activity was detected using the Dual-Luciferase Reporter Assay Kit (Promega, USA) on an iMark microplate reader (Bio-Rad, USA). Relative luciferase activity was calculated and normalized to the activity of *Renilla* luciferase. At least three biological replicates were set up for each experiment.

### Statistical analysis

All statistical analyses were conducted using GraphPad Prism 9.3 and SPSS 23.0, and measurement data that conformed to a normal distribution were presented as the mean ± SD. Unpaired t-tests were conducted to determine the differences between two groups of data that followed a normal distribution. For data consistent with homogeneity of variance, the differences among multiple groups were determined by conducting one-way ANOVA with multiple comparisons using the Bonferroni method. Count data were expressed as the number of cases (n). The clinical features (e.g., TNM stage, lymph node metastasis, and tumor size) were analyzed as categorical variables by performing the Chi-square test. Spearman’s rank correlation was performed for correlation analysis between the hsa_circ_0006837 and miR-424-5p expression levels. The Kaplan-Meier survival curve was obtained by assessing cumulative survival by conducting the log-rank test. Multivariate Cox analysis was performed to assess the prognostic risk factors affecting GC patients. All results were considered to be statistically significant at *P* < 0.05.

## Results

### Expression and clinical significance of hsa_circ_0006837 in GC

**Fig. 1:**
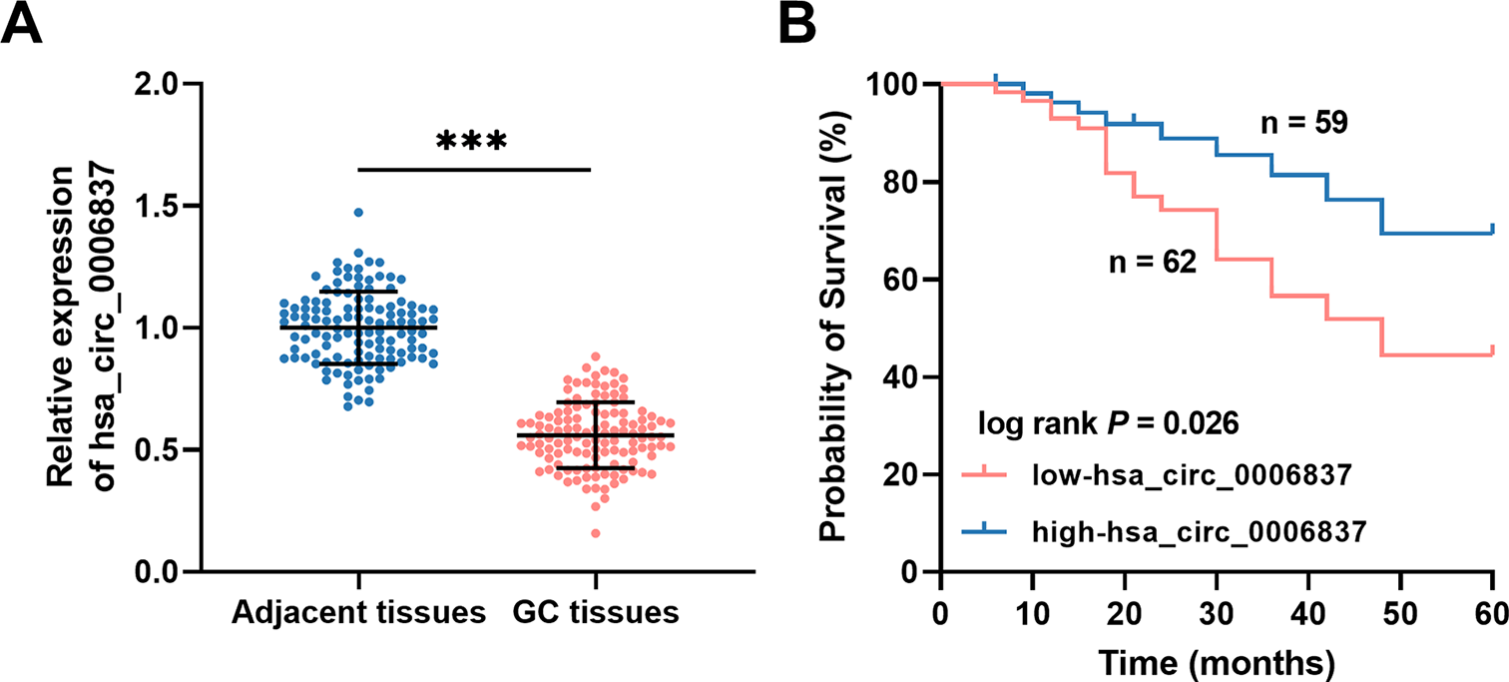
Expression and significance of hsa_circ_0006837 in GC. **(A)** Compared to adjacent normal tissues, the relative expression of hsa_circ_0006837 was decreased in GC tissues. Student’s t-test, ***P < 0.001 (GC tissues vs. adjacent tissues). **(B)** Kaplan-Meier analysis indicated that patients with poorer hsa_circ_0006837 expression had lower survival

The results of the RT-qPCR analysis revealed that the relative expression of hsa_circ_0006837 was considerably lower in GC tissues than in adjacent normal tissues (Fig. 1A). To investigate the significance of the downregulation of hsa_circ_0006837 in GC, clinical data were analyzed in this study, and the results were as follows. First, GC patients were grouped according to the average expression of hsa_circ_0006837 (0.559) in tissues, resulting in 121 GC patients divided into a low-hsa_circ_0006837 group (62 cases) and a high-hsa_circ_0006837 group (59 cases). Second, each clinical feature of GC patients was compared with the varying expression levels of hsa_circ_0006837 in tissues, and the results revealed that hsa_circ_0006837 was strongly related to the TNM stage (*P* = 0.005), lymph node metastasis (*P* = 0.015), and tumor size (*P* = 0.038) of GC patients (Table 1). A Kaplan-Meier curve was plotted to assess the relationship between hsa_circ_0006837 expression and five-year overall survival; the results revealed that patients with lower hsa_circ_0006837 expression exhibited considerably poorer survival than those with higher hsa_circ_0006837 expression (Fig. 1B). Finally, the results of the multivariate regression analysis revealed that hsa_circ_0006837 (HR = 2.901, 95% CI = 1.116–7.542), TNM stage (HR = 2.441, 95% CI = 1.065–5.592), and lymph node metastasis (HR = 2.755, 95% CI = 1.108–6.847; Table 2) were independent prognostic indicators for GC.

**Table 1:** The association between Hsa_circ_0006837 expression and clinical characteristics of GC patients

Characteristics	Cases (*n* = 121)	Hsa_circ_0006837 expression		*P* value
		Low (*n* = 62)	High (*n* = 59)	
Age (years)				0.406
< 60	63	30	33	
≥ 60	58	32	26	
Gender				0.954
Male	68	35	33	
Female	53	27	26	
Lymph node metastasis				0.015
Absent	77	33	44	
Present	44	29	15	
TNM stage				0.005
I-II	75	31	44	
III	46	31	15	
Differentiation				0.106
Well/moderate	71	32	39	
Poor	50	30	20	
Invasion depth				0.228
T1-T2	65	30	35	
T3-T4	56	32	24	
Tumor size (cm)				0.038
< 5	56	23	33	
≥ 5	65	39	26	

**Table 2:** Multivariate analysis of prognostic predictors for overall survival in GC patients

Characteristics	HR factor	95% CI	*P* value
Hsa_circ_0006837	2.901	1.116–7.542	0.029
Age	1.778	0.804–3.934	0.155
Gender	1.501	0.647–3.483	0.344
Lymph node metastasis	2.755	1.108–6.847	0.029
TNM stage	2.441	1.065–5.592	0.035
Differentiation	1.716	0.759–3.882	0.195
Invasion depth	2.130	0.857–5.296	0.104
Tumor size (cm)	1.309	0.574–2.987	0.522

### Effect of hsa_circ_0006837 overexpression on GC cells

**Fig. 2:**
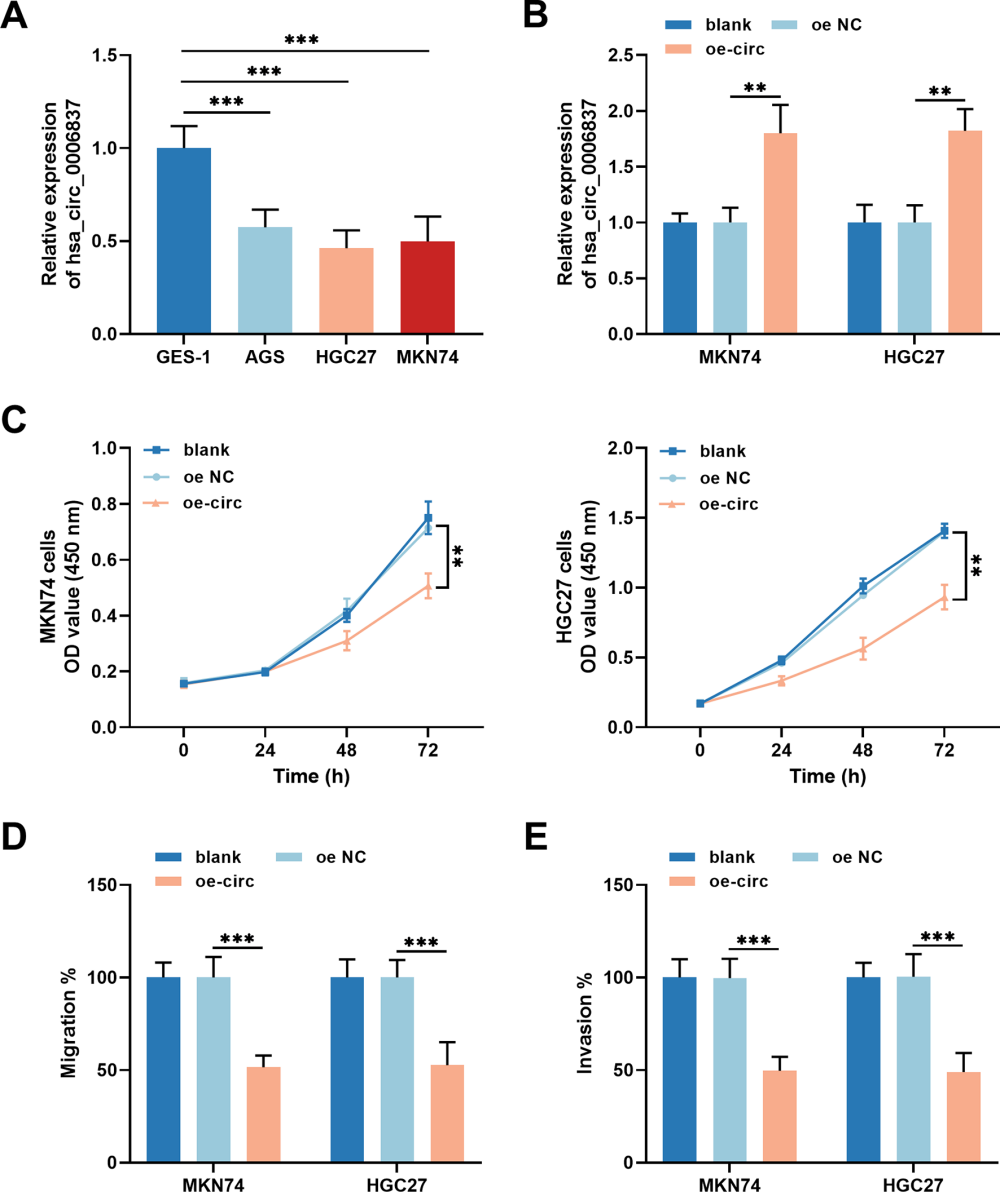
Regulation of GC cellular processes by overexpressing hsa_circ_0006837. **(A)** The expression of hsa_circ_0006837 was much lower in GC cells than in GES-1 cells. Student’s t-test, ***P < 0.001 (AGS, HGC27, MKN74 vs. GES-1). **(B)** Transfection of the oe-circ dramatically upregulated hsa_circ_0006837 in MKN74 and HGC27 cells. **(C)** Overexpression of hsa_circ_0006837 suppressed MKN74 and HGC27 cell proliferation. **D-E.** Hsa_circ_0006837 overexpression depressed MKN74 and HGC27 cell migration (**D**) and invasion (**E**) ability. One-way ANOVA, **P < 0.01, ***P < 0.001 (oe-circ vs. oe NC)

Consistent with the downregulation of hsa_circ_0006837 in GC tissues, a decrease in the expression of hsa_circ_0006837 was also detected in different GC cell lines (Fig. 2A). Owing to these results, MKN74 and HGC27 cells were randomly selected for the following experiments. Compared to NC transfection, oe-circ transfection increased the expression of hsa_circ_0006837 in MKN74 and HGC27 cells (Fig. 2B). The results of the cell viability assay indicated that overexpression of hsa_circ_0006837 repressed the proliferation of MKN74 and HGC27 cells (Fig. 2C). Moreover, the inhibitory effects of the overexpression of hsa_circ_0006837 on the migration (Fig. 2D) and invasion (Fig. 2E) of MKN74 and HGC27 cells were confirmed by conducting Transwell assays.

### Hsa_circ_0006837 regulated GC cell progression via miR-424-5p

**Fig. 3:**
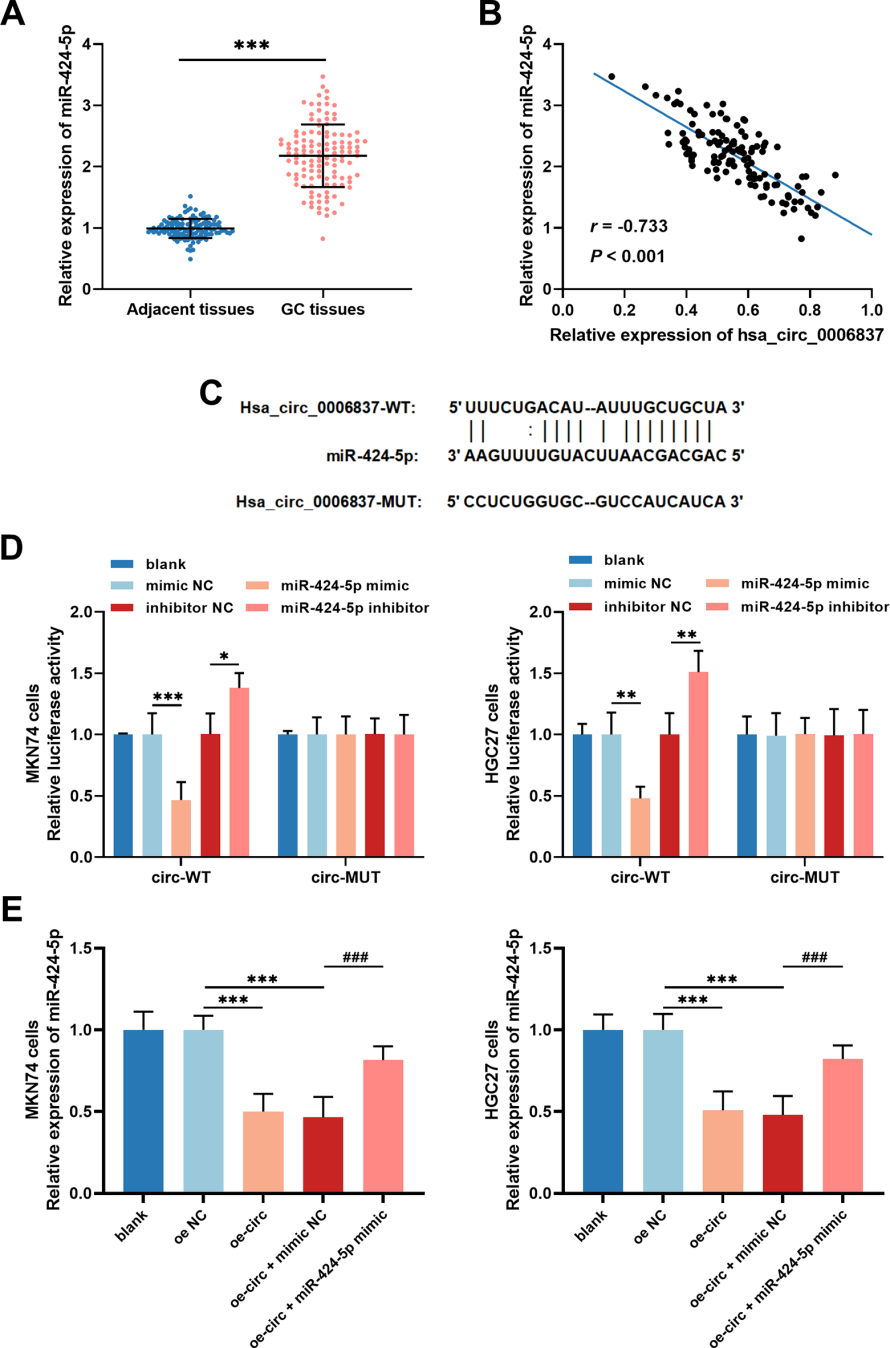
Hsa_circ_0006837 negatively modulated miR-424-5p in a targeted manner. **(A)** Compared to adjacent normal tissues, miR-424-5p was relatively upregulated in GC tissues. Student’s t-test, ***P < 0.001 (GC tissues vs. adjacent tissues). **(B)** The levels of hsa_circ_0006837 and miR-424-5p in GC tissues were negatively correlated. **(C)** Possible binding sites for miR-424-5p and hsa_circ_0006837 were predicted by the starBase database. **(D)** The luciferase activity of circ-WT was repressed by upregulated miR-424-5p and increased by downregulated miR-424-5p. One-way ANOVA, *P < 0.05, **P < 0.01, ***P < 0.001 (miR-424-5p mimic vs. mimic NC, miR-424-5p inhibitor vs. inhibitor NC). **(E)** miR-424-5p expression was downregulated by hsa_circ_0006837 overexpression, which was partially restored by miR-424-5p mimic. One-way ANOVA, ***P < 0.001 (oe-circ or oe-circ + mimic NC vs. oe NC); ^###^*P* < 0.001 (oe-circ + miR-424-5p mimic vs. oe-circ + mimic NC)

**Fig. 4:**
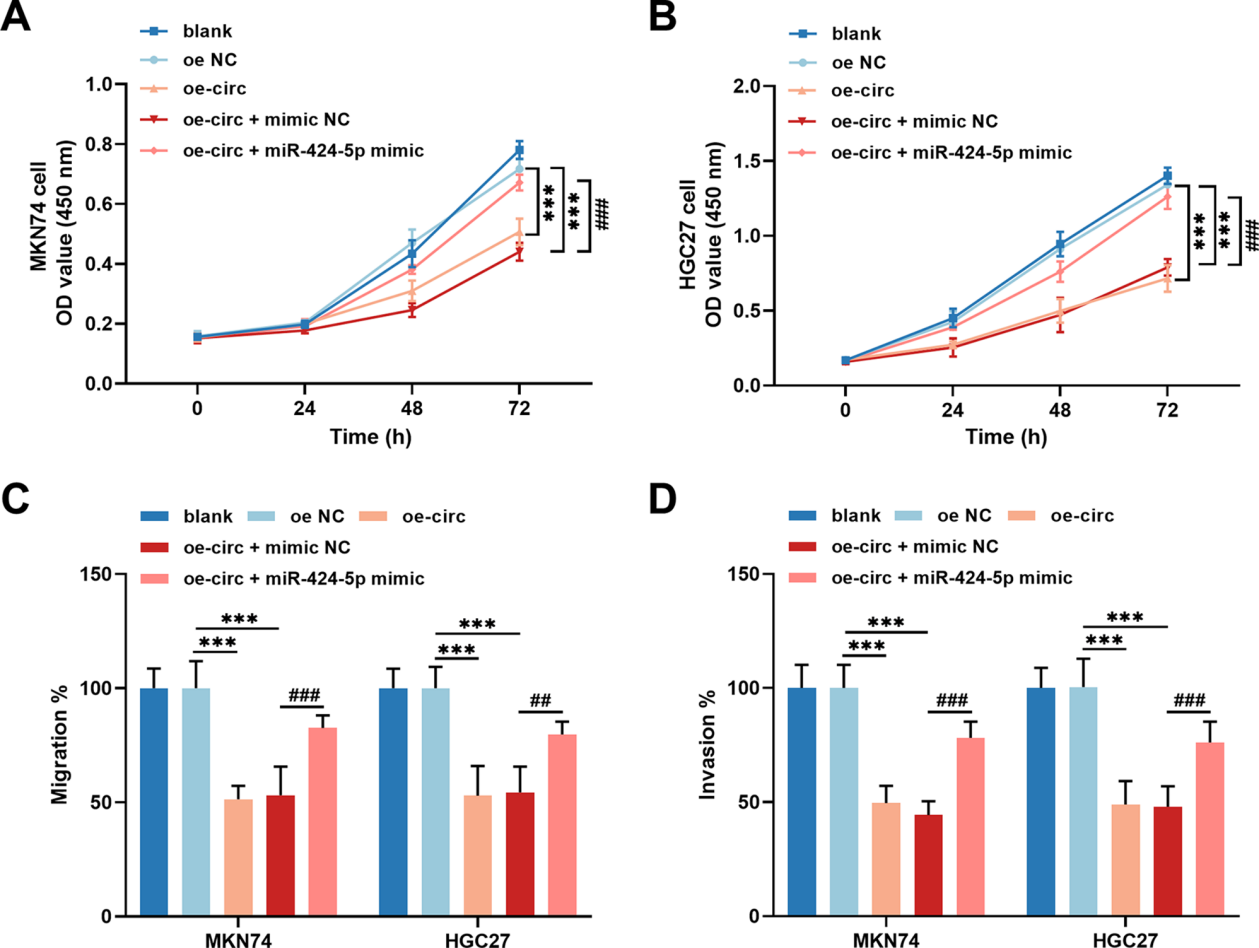
Hsa_circ_0006837 modulated GC cellular processes through modulation of miR-424-5p. **A-B.** Overexpression of hsa_circ_0006837 suppressed cell growth of MKN74 (**A**) and HGC27 (**B**) cells, which was attenuated by miR-424-5p. **C-D.** The migration (**C**) and invasion (**D**) of MKN74 and HGC27 cells were repressed by hsa_circ_0006837 overexpression, and the inhibitory effect was alleviated by miR-424-5p. One-way ANOVA, ***P < 0.001 (oe-circ or oe-circ + mimic NC vs. oe NC); ^##^*P* < 0.01, ^###^*P* < 0.001 (oe-circ + miR-424-5p mimic vs. oe-circ + mimic NC)

In the GC tissue samples, miR-424-5p expression was upregulated (Fig. 3A) and showed a strong negative correlation with the expression of hsa_circ_0006837 (*r* = − 0.733, Fig. 3B). As predicted by the starBase database, miR-424-5p may have binding sites to bind with hsa_circ_0006837 (Fig. 3C). In GC cells, upregulation of miR-424-5p strongly suppressed the activity of circ-WT luciferase, whereas downregulation of miR-424-5p increased this luciferase activity (Fig. 3D). Additionally, downregulation of miR-424-5p was detected in MKN74 and HGC27 cells transfected with oe-circ, which could be rescued by the miR-424-5p mimic (Fig. 3E). Moreover, the overexpression of miR-424-5p attenuated the decrease in MKN74 (Fig. 4A) and HGC27 (Fig. 4B) cell proliferation caused by oe-circ. Furthermore, miR-424-5p alleviated the inhibition of MKN74 and HGC27 cell migration (Fig. 4C) and invasion (Fig. 4D) by the overexpression of hsa_circ_0006837.

### FBXO21 was a downstream gene of miR-424-5p

**Fig. 5:**
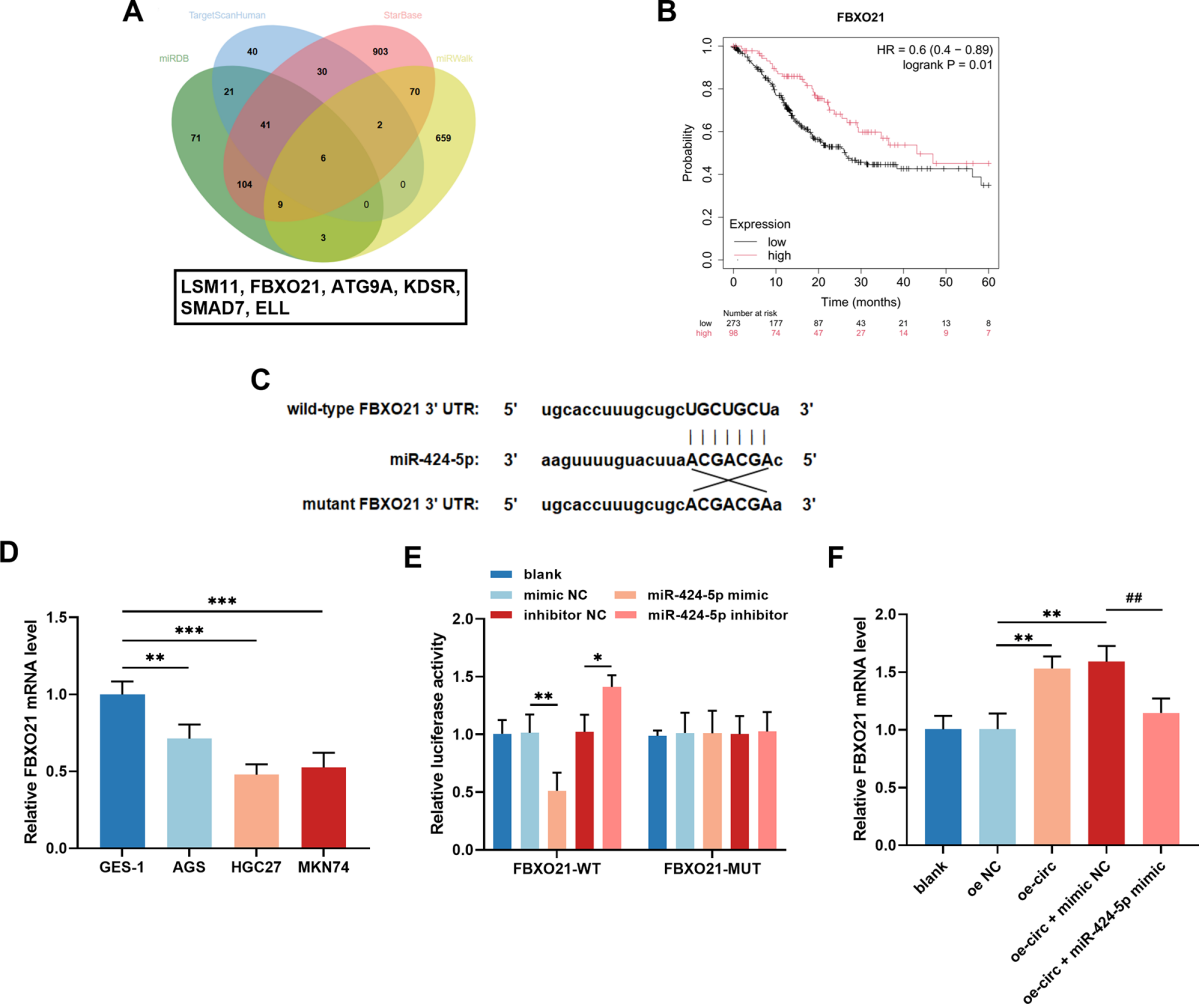
FBXO21 was a downstream gene of the hsa_circ_0006837/miR-424-5p axis. **(A)** The target genes of miR-424-5p were predicted by TargetScanHuman, starBase, miRDB, and miRWalk online databases. **(B)** FBXO21 expression was shown to be related to patient survival in the Kaplan-Meier Plotter database. **(C)** The predicted binding sites of miR-424-5p and FBXO21. **(D)** The level of FBXO21 was decreased in GC cells compared to GES-1 cells. Student’s t-test, **P < 0.01, ***P < 0.001 (AGS, HGC27, MKN74 vs. GES-1). **(E)** In HGC27 cells, miR-424-5p regulated the luciferase activity of FBXO21-WT. One-way ANOVA, *P < 0.05, **P < 0.01 (miR-424-5p mimic vs. mimic NC, miR-424-5p inhibitor vs. inhibitor NC). **(F)** Hsa_circ_0006837 regulated FBXO21 mediated by miR-424-5p. One-way ANOVA, **P < 0.01 (oe-circ or oe-circ + mimic NC vs. oe NC); ^##^*P* < 0.01 (oe-circ + miR-424-5p mimic vs. oe-circ + mimic NC)

Six potential targets of miR-424-5p were predicted using the TargetScanHuman, starBase, miRDB, and miRWalk databases (Fig. 5A). A Kaplan-Meier plot revealed that only FBXO21, ATG9A, and KDSR were significantly correlated with the prognosis of patients with GC (*P* < 0.05, Figure S2). Analysis using the Kaplan-Meier plotter database revealed that GC patients with low expression of FBXO21 had a lower survival rate (*P* = 0.01, Fig. 5B). Subsequent studies revealed that the transfection of GC cells with the miR-424-5p inhibitor considerably upregulated the expression of FBXO21, suggesting a specific regulatory relationship (Figure S3). Moreover, the expression of FBXO21 in GC tissues was positively correlated with that of hsa_circ_0006837 (*r* = 0.712, Figure S4).

**Fig. 6:**
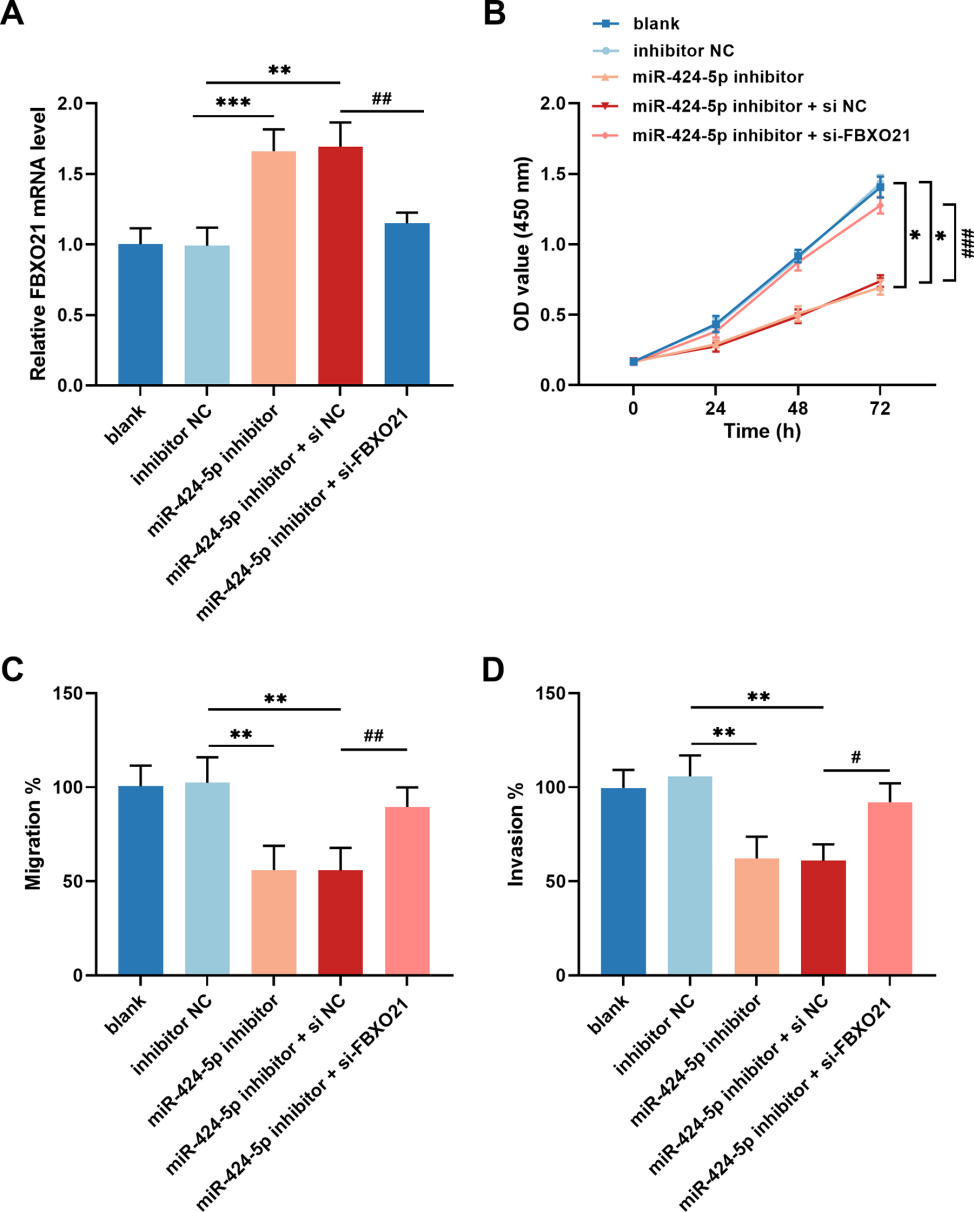
The function of miR-424-5p/FBXO21 axis in GC cells. **(A)** FBXO21 expression exhibited an increase in response to decreased miR-424-5p expression, and the knockdown of FBXO21 led to the reversal of this effect. **(B)** Downregulation of miR-424-5p suppressed the growth of HGC27 cells, and knockdown of FBXO21 attenuated this inhibition. **C-D.** Low expression of miR-424-5p repressed the migration (**C**) and invasion (**D**) of HGC27 cells, while silencing of FBXO21 alleviated the inhibitory effect. One-way ANOVA, *P < 0.05, **P < 0.01, ***P < 0.001 (miR-424-5p inhibitor or miR-424-5p inhibitor + si NC vs. inhibitor NC); ^#^*P* < 0.05, ^##^*P* < 0.01, ^###^*P* < 0.001 (miR-424-5p inhibitor + si-FBXO21 vs. miR-424-5p inhibitor + si NC)

The binding sites of FBXO21 and miR-424-5p were predicted using the TargetScanHuman database (Fig. 5C). Furthermore, in vitro experiments confirmed that FBXO21 is a downstream gene of miR-424-5p. The expression of FBXO21 was dramatically reduced in GC cells (Fig. 5D). The miR-424-5p mimic decreased the luciferase activity of FBXO21-WT in HGC27 cells, and the miR-424-5p inhibitor had the opposite effect. No notable effect of miR-424-5p on the luciferase activity of FBXO21-MUT was detected (Fig. 5E). The overexpression of hsa_circ_0006837 increased FBXO21 mRNA levels, which was reversed by the miR-424-5p mimic (Fig. 5F). In HGC27 cells, FBXO21 expression increased due to decreased miR-424-5p expression; knocking down FBXO21 reversed this effect (Fig. 6A). Moreover, the downregulation of miR-424-5p hindered the growth (Fig. 6B), migration (Fig. 6C), and invasion (Fig. 6D) of HGC27 cells, which was mitigated by the reduction in the expression of FBXO21.

## Discussion

With the emergence of immunotherapy, targeted therapy, and novel therapeutic agents, the landscape of GC treatment has changed. However, owing to the high heterogeneity of tumors, the overall prognosis is unsatisfactory. Metastasis, relapse, and drug resistance are the main reasons for treatment failure and death in GC patients [22, 23]. Abnormal expression of circRNAs has prospective applications in the diagnosis, targeted therapy, and prognostic evaluation of GC. For example, several circRNAs, such as hsa_circ_0086720, hsa_circ_0001020, and hsa_circ_0015286, are useful for screening GC and predicting patient prognosis [24–26]. Recently, hsa_circ_0005480 was reported to be upregulated in patients with GC and could act as a biomarker for the diagnosis and monitoring of prognosis [27]. Hsa_circ_0006837 was identified as a downregulated circRNA based on its expression profile in GC [12]. In this study, the specific role of hsa_circ_0006837 in GC was assessed. Hsa_circ_0006837 was downregulated in GC tissues, which is consistent with the findings of previous studies. Clinically, hsa_circ_0006837 was found to be strongly correlated with crucial indicators of GC progression and severity, such as TNM stage and lymph node metastasis. Moreover, hsa_circ_0006837 can serve as an independent predictor of prognosis and is closely associated with the overall survival of patients with GC. Previously, circRERE (hsa_circ_0009581) was found to assist in predicting the prognosis of patients with multiple myeloma [13]. The above findings indicated that RERE-derived hsa_circ_0006837 is a promising prognostic biomarker and may be involved in the development of GC.

Multiple lines of evidence have confirmed that circRNAs modulate various cellular processes, such as proliferation, apoptosis, and metastasis, in malignant tumors, including GC [28]. A recent study demonstrated a positive effect of hsa_circ_0002019 on the regulation of GC progression [29]. In another study, circRERE (hsa_circ_0009582) was found to promote the growth and invasion of hepatocellular carcinoma cells [30]. Additionally, circRERE (hsa_circ_12952) can decrease colorectal cancer cell proliferation in vitro and restrict tumor growth in vivo [14]. The present study investigated the impact of altered hsa_circ_0006837 expression on the malignant behavior of GC cell lines. Hsa_circ_0006837 overexpression repressed the cellular processes of GC cells, including proliferation, migration, and invasion, which indicated the suppressive effect of hsa_circ_0006837 on GC progression. Therefore, the mechanism of action of hsa_circ_0006837 in GC was investigated next.

CircRNAs function by indirectly regulating downstream gene expression and function, acting as molecular sponges for miRNAs [31]. Several studies have shown that RERE-derived circRNAs can target a wide range of miRNAs to exert effects on various diseases, including multiple myeloma and chronic obstructive pulmonary disease [32, 33]. For example, circRERE (hsa_circ_0009581) was found to interact with miR-152-3p to increase resistance to bortezomib in multiple myeloma [32]. Moreover, circRERE (hsa_circ_12952) can modulate the type I interferon signaling pathway via miR-6837-3p in colorectal cancer [14]. In this study, the negative regulatory role of hsa_circ_0006837 on miR-424-5p in GC was discovered. The miRNA miR-424-5p can mediate the modulation of downstream genes by circRNAs in the progression of hepatocellular carcinoma, endometrial cancer, and oral squamous cell carcinoma [34–36]. Furthermore, miR-424-5p can also repress the growth of GC by the lncRNA MBNL1-AS1 [15]. Here, miR-424-5p was shown to attenuate the suppression of GC cell growth, migration, and invasion caused by the overexpression of hsa_circ_0006837. These findings suggested that hsa_circ_0006837 regulates GC progression via the modulation of miR-424-5p. Researchers have demonstrated the involvement of miR-424-5p in the progression of GC related to the TGF-β pathway [15, 37], providing a foundation for subsequent studies. In the context of tumor progression, the TGF-β signaling pathway can act as a promoter of tumor cell growth. For example, it can achieve immunosuppression by inhibiting the proliferation and function of some immune cells (e.g., T cells) [38]. It can also induce the formation of a microenvironment that is favorable for tumor metastasis and facilitates epithelial-mesenchymal transition in tumors [39]. Exploring whether the hsa_circ_0006837/miR-424-5p axis is involved in the TGF-β signaling pathway would be beneficial in clarifying its key role in GC occurrence and development.

In this study, FBXO21 expression was positively correlated with hsa_circ_0006837 expression in GC tissues. We also identified FBXO21 as a downstream target of miR-424-5p. In vitro experiments demonstrated that FBXO21 was modulated by hsa_circ_0006837 and miR-424-5p. Previously, low FBXO21 expression was reported to be associated with a poor prognosis in patients with GC, and FBXO21 was found to inhibit the progression of GC by suppressing proliferation and epithelial-to-mesenchymal transition [40]. In this study, miR-424-5p was observed to exert a negative regulatory effect on the expression of FBXO21. Subsequent experiments demonstrated that the miR-424-5p/FBXO21 axis modulates the malignant behavior of GC cells, which may represent a key mechanism underlying the progression of GC. In vivo experiments are indispensable for studying GC-related molecular mechanisms. The findings of this study need to be validated by conducting animal experiments (e.g., xenograft models) to further reveal the effects of hsa_circ_0006837 on the growth of GC in vivo.

The principal finding of this study was that hsa_circ_0006837 exerts its regulatory effect on the progression of GC via miR-424-5p. However, the observed inverse correlation between hsa_circ_0006837 and miR-424-5p may not be solely due to the sponge effect, and the potential contribution of miRNA-mediated circRNA decay (via AGO proteins) should also be considered, which deserves further exploration. The potential interaction or downstream pathways of hsa_circ_0006837 with other miRNAs may have been overlooked. Further investigations are needed to elucidate whether hsa_circ_0006837 exerts regulatory effects on other miRNAs in GC. Although circRNAs have been observed to exhibit differential expression under a range of pathological conditions, further investigation is needed to elucidate the specific mechanisms and specificity of their involvement. Comparing the expression levels of hsa_circ_0006837 with those of other known circRNAs and assessing their correlation with GC prognosis may provide valuable information. Moreover, the implementation of circRNAs in clinical diagnosis and therapy necessitates comprehensive clinical validation, including independent large sample sets and high-throughput sequencing technologies.

To summarize, hsa_circ_0006837 is considerably downregulated in GC and can act as a biomarker for the prognosis and progression of GC. The overexpression of hsa_circ_0006837 could modulate the malignant behaviors of GC cells through the miR-424-5p/FBXO21 axis. This study provided an experimental basis for the application of hsa_circ_0006837 in the prognostic assessment and treatment of GC and revealed new strategies for personalized treatment.

## Electronic supplementary material

Below is the link to the electronic supplementary material.

Supplementary Material 1

## Data Availability

The datasets used and/or analysed during the current study are available from the corresponding author on reasonable request.
