# Supplementary material for: Hsa_circ_0006837 suppresses gastric cancer cell proliferation, migration, and invasion via the modulation of miR-424-5p
Source: Hereditas. 2025 May 22;162:85. doi: 10.1186/s41065-025-00449-w (PMC12100860; doi:10.1186/s41065-025-00449-w)
Supplement: Supplementary file 1 — Supplementary Material 1 [file 41065_2025_449_MOESM1_ESM.docx]

>hsa_circ_0006837|NM_012102|RERE

AGTAAGAGGGACCATCTCCTCATGAACGTCAAATGGTACTACCGTCAATCTGAGGTTCCAGATTCTGTGTATCAGCATTTGGTTCAGGATCGACATAATGAAAATGACTCTGGAAGAGAACTTGTCATTACAGACCCAGTTATCAAGAACCGAGAGCTCTTCATTTCTGATTACGTTGACACTTACCATGCTGCTGCCCTTAGAGGGAAGTGTAACATCTCCCATTTTTCTGACATATTTGCTGCTAGAGAGTTTAAAGCCCGAGTGGATTCATTTTTCTACATATTAGGATATAACCCTGAGACAAGGAGGCTGAACAGTACCCAGGGGGAGATTCGTGTCGGTCCTAGTCATCAGGCCAAACTTCCAGATCTGCAACCATTTCCTTCTCCAGATGGTGATACAGTGACCCAACATGAGGAACTGGTCTGGATGCCTGGAGTTAACGACTGTGACCTCCTTATGTACTTGAGGGCAGCAAGGAGCATGGCGGCATTTGCAGGAATGTGTGATGGAGGCTCTACAGAGGACGGCTGTGTCGCAGCCTCTCGGGATGACACCACTCTGAATGCACTGAACACA


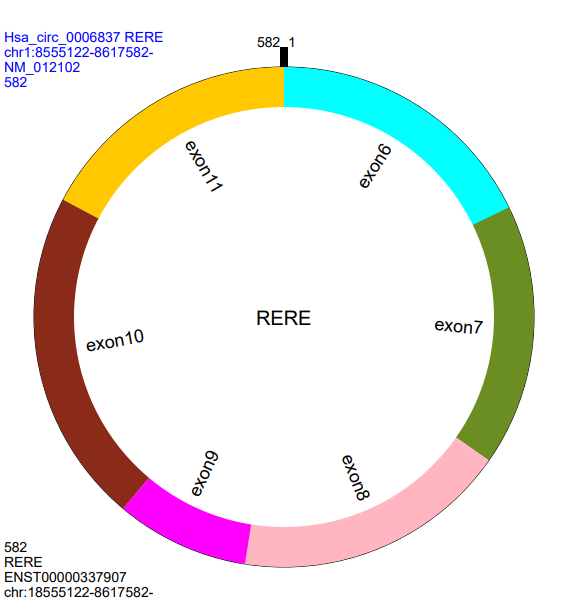


**Figure S1.** The spliced sequence and structure of hsa_circ_0006837.


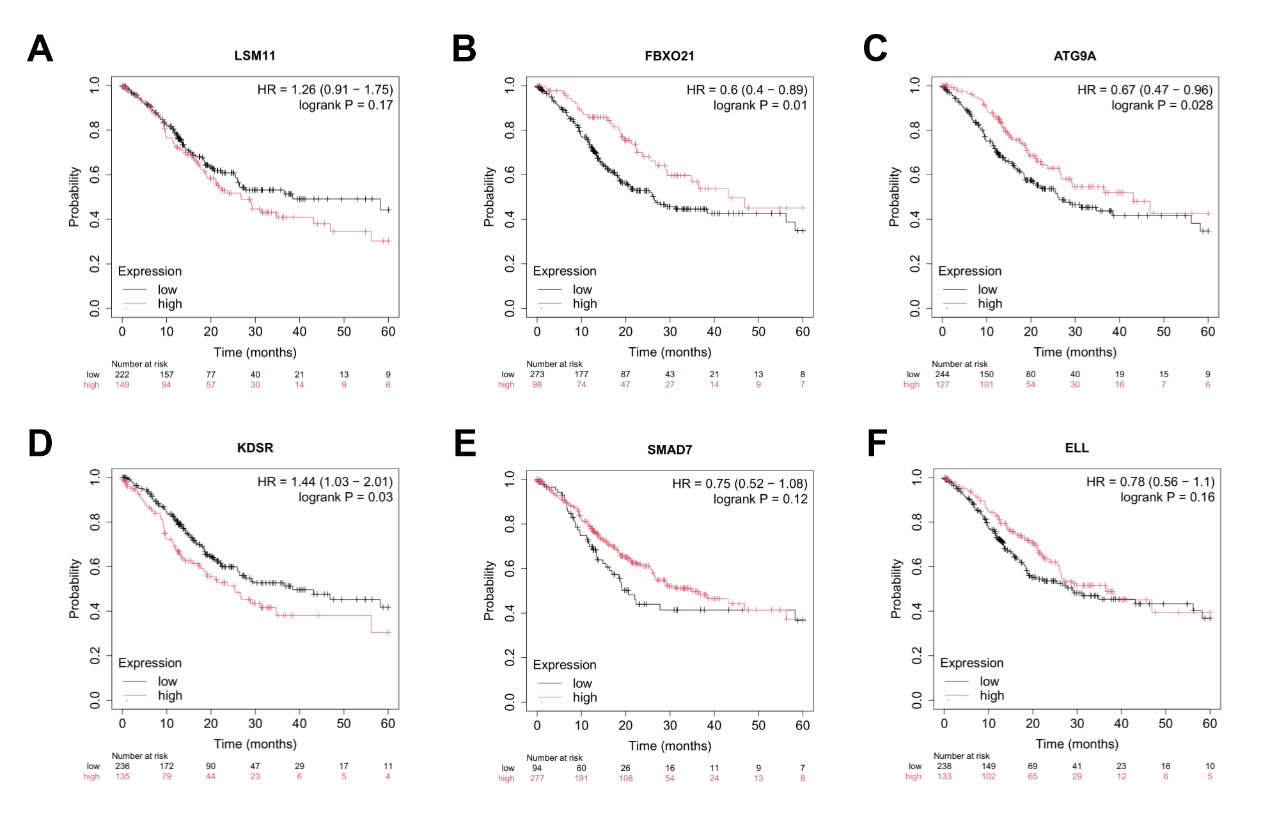


**Figure S2.** The association of predicted genes with GC prognosis was analyzed by bioinformatics methods. Kaplan-Meier curves of LSM11 (**A**), FBXO21 (**B**), ATG9A (**C**), KDSR (**D**), SMAD7 (**E**), and ELL (**F**) in the online database Kaplan-Meier Plotter.


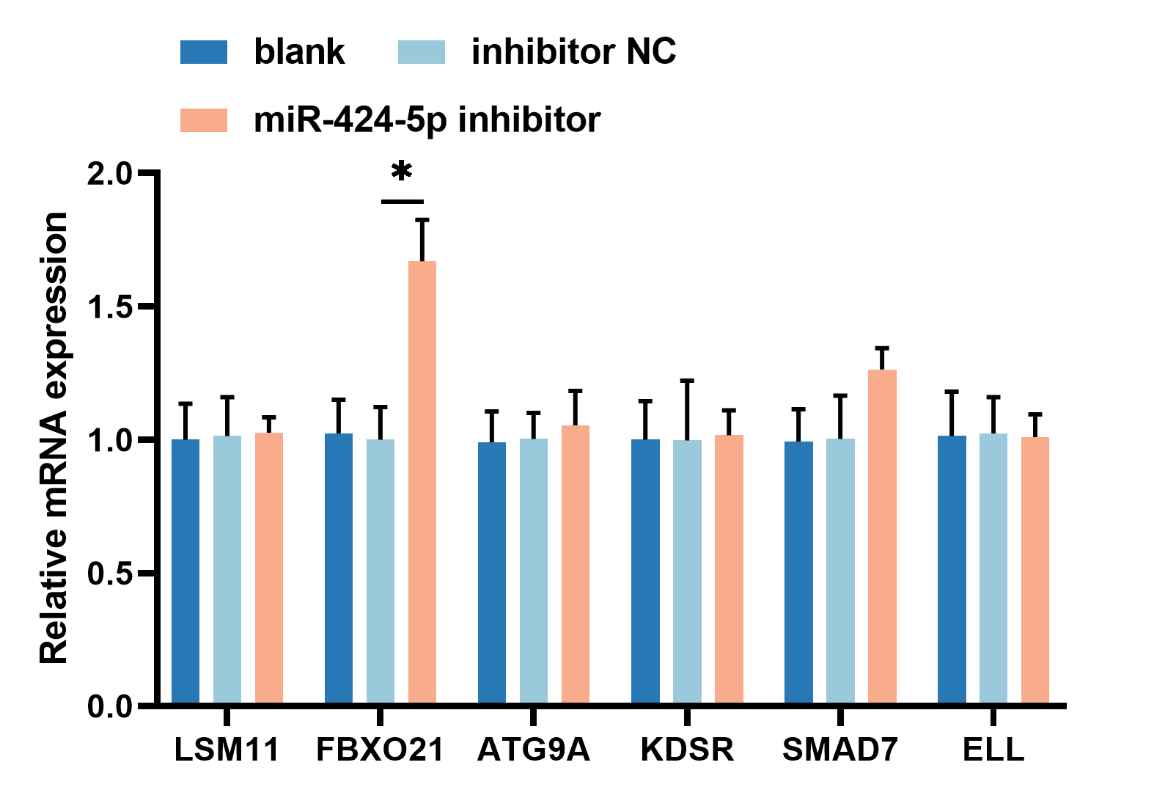


**Figure S3.** Effect of miR-424-5p inhibitor on mRNA levels of six predicted genes.


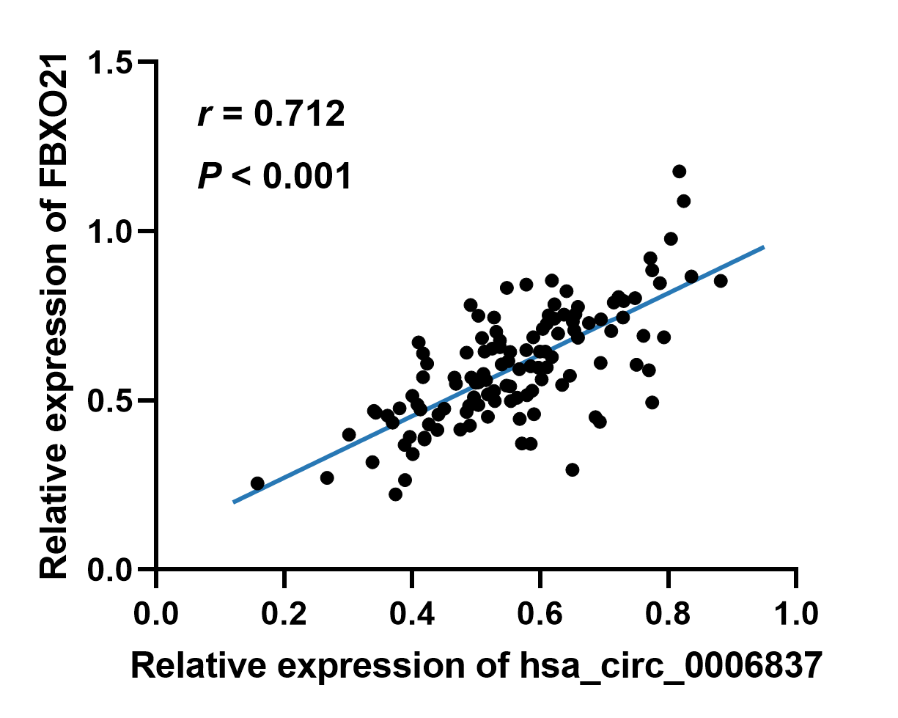


**Figure S4.** Correlation of hsa_circ_0006837 and FBXO21 expression in GC tissues.

**Table S1.** Primer sequences for plasmid construction.

| Plasmid name | Sequence (5'-3') | Position |
| --- | --- | --- |
| Oe-hsa_circ_0006837 | F: TACCGAGCTCGGATCCTATAACCCTGAGACAAGGAGG | *BamH* I |
|  | R: GCCCTCTAGACTCGAGTCCTAATATGTAGAAAAATGAATCC | *Xho* I |
| Hsa_circ_0006837-WT | F: TTTCTCTATCGATAGGTACCTATAACCCTGAGACAAGGAGG | *Kpn* I |
|  | R: CCGGAATGCCAAGCTTTCCTAATATGTAGAAAAATGAATCC | *Hind* III |
| Hsa_circ_0006837-MUT | F: TCTGGTGCGTCCATCATCAGAGAGTTTAAAGCCCGAGTGG |  |
|  | R: GATGGACGCACCAGAGGAATGGGAGATGTTACACTTCCC |  |
| Wild-type FBXO21 3' UTR | F: GTACTTGGAGCGGCCGCTGGAGCCTCAAGAAATCTCACACC | *Not* I |
|  | R: GATCGCAGATCTCGAGGACAAGCATACAAGCCATGCC | *Xho* I |
| Mutant FBXO21 3' UTR | F: TTTGCACGACGAGCTATCTTCCAAGAGAACGGGACT |  |
|  | R: AGCTCGTCGTGCAAAGGTGCAATGTCCTCTCT |  |

**Table S2.** Primer sequences for RT-qPCR assays.

| Primer name | Sequence (5'-3') |
| --- | --- |
| Hsa_circ_0006837 | F: GATTCGTGTCGGTCCTAGTCATCAG |
|  | R: CAAGTACATAAGGAGGTCACAGTCGTTA |
| miR-424-5p | F: GCGCAGCAGCAATTCATGT |
|  | R: AGTGCAGGGTCCGAGGTATT |
| FBXO21 | F: GGTCAATGTCAAGAAGGTGTTACAGAGA |
|  | R: TGCCAGATAGAGATCCAGCGAGTC |
| LSM11 | F: AACTCCATCGCTGTATCCGTGAG |
|  | R: ACATCAGTAAGTGCCATATTCCAGAAC |
| ATG9A | F: CATCCTGGTCATTGCTGGTGTCTTC |
|  | R: TCTGGTGCTCCTTCTGCGTCTG |
| KDSR | F: GCCTTCGTGCTGCTGCTGTA |
|  | R: GCCACTGGAACCTCCTGTAACC |
| SMAD7 | F: TCGGAAGTCAAGAGGCTGTGTTG |
|  | R: TAGTTCGCAGAGTCGGCTAAGGT |
| ELL | F: GTTAGCGACGGCAGCAAGGT |
|  | R: CGATGTTGGAGAGGTAGAAGGAGAAC |
| β-actin | F: ACCCAGCACAATGAAGATCA |
|  | R: CGTCATACTCCTGCTTGCTG |
| U6 | F: CTCGCTTCGGCAGCACA |
|  | R: AACGCTTCACGAATTTGCGT |
